# Cannabis use, decision making, and perceptions of risk among breastfeeding individuals: the Lactation and Cannabis (LAC) Study

**DOI:** 10.1186/s42238-023-00212-w

**Published:** 2024-02-16

**Authors:** Caroline B. Smith, Jenna Schmidt, Elizabeth A. Holdsworth, Beatrice Caffé, Olivia Brooks, Janet E. Williams, David R. Gang, Mark A. McGuire, Michelle K. McGuire, Celestina Barbosa-Leiker, Courtney L. Meehan

**Affiliations:** 1 Department of Anthropology, Washington State University, Pullman, WA USA; 2 Department of Animal, Veterinary, and Food Sciences, University of Idaho, Moscow, ID USA; 3 Institute of Biological Chemistry, Washington State University, Pullman, WA USA; 4 Margaret Ritchie School of Family and Consumer Sciences, University of Idaho, Moscow, ID USA; 5 College of Nursing, Washington State University Health Sciences Spokane, Spokane, WA USA

**Keywords:** Marijuana, Substance use, Breastfeeding, Human milk, Perinatal

## Abstract

**Objective:**

Our primary objective was to understand breastfeeding individuals’ decisions to use cannabis. Specifically, we investigated reasons for cannabis use, experiences with healthcare providers regarding use, and potential concerns about cannabis use.

**Methods:**

We collected survey data from twenty breastfeeding participants from Washington and Oregon who used cannabis at least once weekly. We documented individuals’ cannabis use and analyzed factors associated with their decisions to use cannabis during lactation. Qualitative description was used to assess responses to an open-ended question about potential concerns.

**Results:**

Fifty-five percent of participants (*n* = 11) reported using cannabis to treat or manage health conditions, mostly related to mental health. Eighty percent of participants (*n* = 16) reported very few or no concerns about using cannabis while breastfeeding, although participants who used cannabis for medical purposes had significantly more concerns. Most participants (*n* = 18, 90%) reported receiving either no or unhelpful advice from healthcare providers. Four themes arose through qualitative analysis, indicating that breastfeeding individuals are: 1) identifying research gaps and collecting evidence; 2) monitoring their child’s health and development; 3) monitoring and titrating their cannabis use; and 4) comparing risks between cannabis and other controlled substances.

**Conclusions:**

Breastfeeding individuals reported cannabis for medical and non-medical reasons and few had concerns about cannabis use during breastfeeding. Breastfeeding individuals reported using a variety of strategies and resources in their assessment of risk or lack thereof when deciding to use cannabis. Most participants reported receiving no helpful guidance from healthcare providers.

## Introduction

Research on cannabis use during lactation and its impact, or lack thereof, on infant outcomes is exceedingly limited (Ryan et al. 2018; Metz and Stickrath 2015). To avoid potential harm in the absence of information, the American College of Obstetrics and Gynecologists, the Academy of Breastfeeding Medicine, and the American Academy of Pediatrics encourage reducing or ceasing cannabis use during lactation (Ryan et al. 2018; Committee on Obstetric Practice 2017; Reece-Stremtan et al. 2015). The Centers for Disease Control and Prevention state that breastfeeding mothers should be advised against using cannabis while noting that data are insufficient to determine whether or not it is safe for mothers who use marijuana to breastfeed (CDC 2022). Despite these recommendations, postpartum cannabis use may be increasing in the United States (US). Postpartum maternal cannabis use increased significantly in US states that legalized cannabis: women residing in states where cannabis has been legalized were 1.83 times more likely to use cannabis during the postpartum period than women residing in states where cannabis was not legal (Skelton et al. 2021). Among pregnant women in California, frequency of cannabis use increased significantly from 2009 to 2017, with daily use increasing most rapidly (from 0.28% to 0.69%) (Young-Wolff et al. 2019). Moreover, perceptions among reproductive-aged women that cannabis poses no risk increased from 4.6% in 2005 to 19.0% in 2015 (Jarlenski et al. 2017). Likewise, pregnant and perinatal women often perceive that cannabis use can offer benefits and can be safe (Barbosa-Leiker et al. 2020; Bayrampour et al. 2019; Chang et al. 2019; Kiel et al. 2023). Estimates of breastfeeding individuals’ cannabis use in the US are limited, but one study using data from 2014–2015 placed rates at approximately 5 percent (Crume et al. 2018; cf Wang 2017). It is possible, however, that stigma associated with cannabis use during breastfeeding leads to underreporting and underestimates of the rate.

Potential risks to infants from exposure to biologically active compounds in cannabis via human milk are typically inferred from the pregnancy literature, where evidence suggests that Δ9-tetrahydrocannabinol (Δ9-THC)—the main psychoactive component in cannabis—crosses the placenta (Grant et al. 2018). While results of previous systematic reviews were mixed (Conner et al. 2016; Gunn et al. 2016), a recent systematic review found increased risk of adverse outcomes associated with prenatal cannabis use (Baía and Domingues 2022). Risks included low birth weight, preterm birth, and small size for gestational age while controlling for tobacco and other illicit drug use. There is also some evidence to suggest that prenatal cannabis exposure is associated with long-term negative impacts on child development, including greater risk for psychopathology and problems with executive functioning (Grandy et al. 2022; Joseph and Vettraino 2020).

Research on maternal cannabis use while breastfeeding and infant health outcomes is even more limited. Nevertheless, evidence from pharmacokinetic studies suggests that Δ9-THC passes into human milk (Baker et al. 2018). In one study, Δ9-THC was detectable in a human milk sample 6 days after last reported maternal cannabis use (Bertrand et al. 2018), while a longitudinal study estimated the half-life of Δ9-THC in human milk to be 17 days, with a projected time to elimination greater than 6 weeks (Wymore et al. 2021). One chronic, heavy user’s milk-to-plasma Δ9-THC ratio was 8:1 (Perez-Reyes and Wall 1982), indicating that Δ9-THC may accumulate in the mammary gland. Furthermore, cannabinoid metabolites were found in a breastfeeding infant’s feces (Perez-Reyes and Wall 1982) and in the urine of children who consumed milk from buffalos who frequently grazed on wild cannabis (Ahmad and Ahmad 1990). These findings indicate that cannabinoids are transferred to infants breastfed by individuals who use cannabis.

Data on infant outcomes of cannabis exposure during breastfeeding are not only limited but conflicting (Seabrook et al. 2017). In the two studies that explicitly examine breastfeeding individuals, one study found reduced motor development in infants nursed by mothers using cannabis at one year of life compared to matched controls (Astley and Little 1990), while the other found no differences in motor or mental skills between cannabis-exposed and unexposed infants at the same age (Tennes et al. 1985). Both studies relied on self-reported cannabis use, and neither could tease apart pre- versus post-natal cannabis exposure. Finally, it is not clear if there are potential harms from cannabis use to infants either through second-hand smoke inhalation or through caregiver impairment (Badowski and Smith 2020).

As a result of the scarcity of research, healthcare professionals cannot confidently point to any results that indicate whether and/or how cannabis use while breastfeeding impacts breastfed infants. This may explain why, despite national guidelines stating that cannabis should not be used during lactation, a study found that only 15% of lactation professionals recommended that mothers cease breastfeeding if they were unable/unwilling to stop using cannabis; 41% recommended continued breastfeeding assuming benefits outweigh risks; and the remaining medical professionals provided variable advice dependent on level of maternal cannabis use (Bergeria and Heil 2015).

There may also be differences in the reasons breastfeeding individuals use cannabis compared to the general population. In a US national sample, 17% of adults who used cannabis during the past year were medical users (defined by having a doctor’s recommendation to use cannabis) (Lin et al. 2016). Rates of medical cannabis use that include self-medication range from 39% in a Canadian sample who used cannabis in the past 6 months (Turna et al. 2020), to 59% of an international cohort (primarily US participants] who used cannabis in the past 30 days (Sexton et al. 2016). In contrast, a survey of women who used cannabis while breastfeeding found that 89% reported using cannabis for health-related reasons (Garner et al. 2022).

The primary objective in this study was to understand breastfeeding individuals’ decision to use cannabis. We addressed this through three main research questions.

1. Do breastfeeding individuals use cannabis to treat health conditions, and if so for which conditions?
2. Do breastfeeding individuals receive guidance on breastfeeding and cannabis use from healthcare providers?
3. Do breastfeeding individuals who use cannabis have concerns about their cannabis use and, in their own words, what factors affect their concerns or lack of concern?

## Methods

Data were collected via surveys between February and May 2022 as part of the Lactation and Cannabis (LAC) Study on breastfeeding, human milk, and cannabinoids. The study was approved by the Washington State University Institutional Review Board, and a National Institutes of Health Certificate of Confidentiality was obtained.

### Participants

We recruited participants through convenience sampling in Washington and Oregon, where legalized medical and non-medical use sales of cannabis to individuals 21 years or older are permitted. We used social and other media advertisements and flyers to recruit breastfeeding women who were ≥ 21 years of age, < 180 days postpartum with a full-term infant (≥ 37 weeks gestation), and who were currently using cannabis at least once per week. Participants needed to breastfeed and/or pump their milk ≥ 5 times daily, and occasionally-to-frequently provide pumped milk to their infants. The last requirement ensured that participation in the parent study (focused on cannabinoid concentrations in human milk), which required collection of multiple milk samples, would not impact infant nutrition and feeding patterns. Infants did not need to be exclusively breastfed. Infants could be fed their parent’s pumped milk and/or provided formula supplementation as long as they met the requirements for pumping/breastfeeding and providing their infant human milk. Exclusion criteria included use of illicit drugs; use of opioid agonist treatments or unprescribed opioids anytime within the past 5 years; symptoms of infection in the 7 days prior to enrollment; and/or indications of a breast infection. A $100 gift card to a retail store was provided for completing the parent study. Data used in this analysis were derived from the survey participants completed at enrollment. Twenty-three eligible participants were enrolled, but three individuals elected not to complete the study, resulting in an analytic sample of 20 participants.

### Quantitative measures and analysis

Participants completed an online survey through the REDCap (Harris et al. 2009) data collection tool that included questions about demographics, infant diet, breastfeeding behavior, substance use, and the Daily Sessions, Frequency, Age of Onset and Quantity of Cannabis Use Inventory (DFAQ-CU) (Cuttler and Spradlin 2017). The DFAQ-CU contains pictures of cannabis in joint, bud, and loose-leaf forms to facilitate estimates of quantities used. We developed additional questions regarding participants’ cannabis use specifically for this analysis.

1. *Have any of your healthcare providers offered advice or guidance about cannabis use while breastfeeding?* Participants could select “Yes, I found it helpful,” “Yes, but I did not find it helpful,” “No, they never gave advice or guidance,” or “Prefer not to answer.”
2. *Do you have any concerns about using cannabis/marijuana while breastfeeding?* Participants could indicate they had “a lot,” “a few,” “very few,” or “no” concerns or that they “hadn’t thought about it very much.”

We categorized participants’ cannabis use as non-medical use if they responded “No” to the question: “Do you use cannabis to treat or manage any health conditions?” and as medical use if they responded “Yes.” Since participants were recruited from states where non-medical cannabis is legal and medical cannabis cards are not required, we relied on self-report of medical use to categorize participants. Participants in the medical use group were asked what medical/health conditions they used cannabis to treat/manage and could list as many conditions as they wished. We grouped responses into broad illness categories (e.g., “anxiety” and “depression” were categorized as mental health conditions; “migraine,” “back pain,” and “sciatica” were categorized as chronic pain conditions). We analyzed days out of the past 30 they used cannabis, average frequency of use, and level of concerns across medical use versus non-medical use groups via Wilcoxon rank sum tests. The Wilcoxon rank sum test was selected because it requires fewer assumptions about the shapes of underlying population distributions and offers a satisfactory alternative to parametric tests with little loss of statistical power, especially in small samples (Kitchen 2009; Bridge and Sawilowsky 1999). Descriptive statistics include mean and standard deviation for continuous data and percent for categorical data. Statistical analyses were conducted in R version 4.0.2 (R Core Team. R 2021) and significance was declared at *p* < 0.05.

### Qualitative measures and analysis

To understand factors that affected perceived risks of cannabis use during lactation, we asked participants to describe the reasons for their concerns or lack thereof about cannabis use while breastfeeding: *If you like, you can elaborate why you do or do not have concerns about using cannabis while breastfeeding*. Although limited to one open-ended question and not a full semi-structured interview (Barbosa-Leiker et al. 2020), we employed a qualitative description methodology (Bradshaw et al. 2017; Sandelowski 2000, 2010) to systematically assess a little-known phenomenon, choosing to breastfeed while using cannabis. The analysis was an iterative process through which participant responses were distilled into themes. Two authors (CS and JS) independently coded the responses manually as common themes arose from the data and then met three times to discuss. Themes were retained if there was agreement between coders. Themes were further revised during manuscript preparation based on consensus between all authors. We took an inductive thematic approach to saturation per Saunders et al. (2018) and ended the coding process when no new codes or themes emerged from the data.

## Results

### Participant characteristics

Twenty participants between the ages of 21 and 37 years completed the study (Table 1). Mean age was 26.6 ± 3.5 years. Mean time postpartum at enrollment was 94.3 ± 49.8 days. Participants reported a range of 2021 household incomes; 35% (*n* = 7) reported income < $20,000 while 20% (*n* = 4) reported income ≥ $75,000. Educational attainment varied with 30% (*n* = 6) holding a high school diploma or having passed the General Educational Development (GED) exam, 20% (*n* = 4) having some college experience but no degree, and 50% (*n* = 10) holding a technical, vocational, associate's, or bachelor's degree. Half the participants (*n* = 10) were married, nine were never married, and one was divorced. When participants reported more than one occupation, we categorized participants within their non-household occupations if they reported one. Most were homemakers (*n* = 13), one was employed full-time, 5 were employed part-time, and one was a student. Participants were asked "*With which race/ethnicity do you identify?”* and could select all that applied from the following: American Indian or Alaska Native, Asian, Black or African American, Native Hawaiian or other Pacific Islander, White, Hispanic or Latinx, or other. Most (70%) self-identified as White, 5% Black or African American, 5% Hispanic or Latinx, 5% Native Hawaiian or other Pacific Islander, and 15% reported more than one race/ethnicity. Seventeen participants (85%) reported exclusively breastfeeding their infants.

**Table 1:** Demographic and anthropometric characteristics of breastfeeding individuals and their infants included in this study (*N* = 20)

**Characteristic**	Mean (SD); n / N (%)
Age (years)	26.6 (3.5)
Education (highest level completed)	
College graduate (bachelor's degree)	5 / 20 (25%)
High school graduate/GED	6 / 20 (30%)
Some college but no degree	4 / 20 (20%)
Technical/vocation degree or associate degree	5 / 20 (25%)
Marital Status	
Divorced	1 / 20 (5%)
Married	10 / 20 (50%)
Never married	9 / 20 (45%)
2021 Household Income ($/year)	
< 20,000	7 / 20 (35%)
20,000—< 35,000	3 / 20 (15%)
35,000—< 50,000	3 / 20 (15%)
50,000—< 75,000	3 / 20 (15%)
75,000—< 100,000	3 / 20 (15%)
≥ 100,000	1 / 20 (5%)
Total number of births	1.9 (0.8)
Gestational age (weeks)	38.8 (1.2)
Maternal time postpartum at enrollment (days)	94.3 (49.8)
Infant birthweight (kilograms)	3.4 (0.5)
Race/ethnicity	
Black or African American	1 / 20 (5%)
Hispanic or Latinx	1 / 20 (5%)
More than one race	3 / 20 (15%)
Native Hawaiian or other Pacific Islander	1 / 20 (5%)
White	14 / 20 (70%)
Employment Status	
Employed full-time	1 / 20 (5%)
Employed part-time	5 / 20 (25%)
Homemaker/Stay-at-home mom	13 / 20 (65%)
Student	1 / 20 (5%)

### Use of cannabis and other substances

**Fig. 1:**
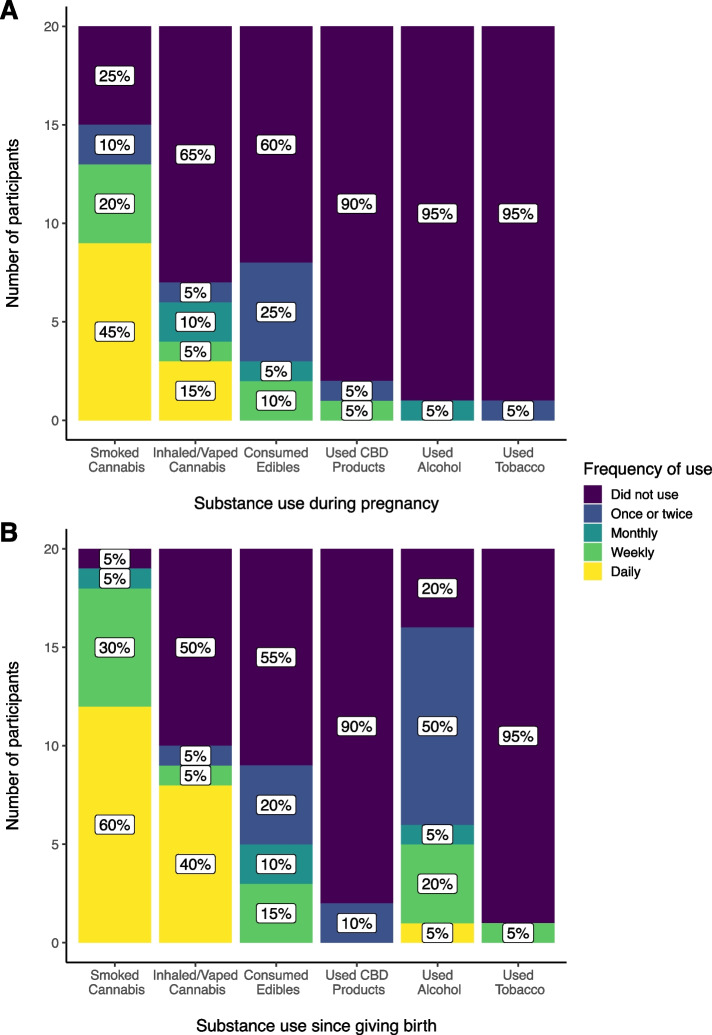
Reported frequency of cannabis, cannabidiol (CBD), alcohol, and tobacco use **a** during pregnancy (*N* = 20) and **b** since giving birth (*N* = 20), by substance type/mode

Sixteen participants (80%) reported using cannabis while pregnant, of which nine reported daily cannabis use during pregnancy (Fig. 1a). One woman reported using tobacco once or twice during pregnancy, and one reported consuming alcohol approximately once a month during pregnancy. No participants reported using heroin, opiates, barbiturates, sedatives, cocaine, amphetamines, hallucinogens, or inhalants during their pregnancies. The same was true in the postpartum period, except for one who reported using a daily stimulant, possibly a prescription. Most participants (*n* = 14, 70%) reported daily cannabis use postpartum, and six (30%) reported weekly use. All but one participant reported smoking cannabis (i.e., using cannabis that has been combusted, for instance in a joint) since giving birth. Half the participants had inhaled or vaped cannabis (i.e., using cannabis that has not been combusted, for instance in a vape pen) since giving birth (Fig. 1b).

On average, participants had used cannabis 23 days out of the past 30 (Table 2). Participants reported using cannabis on average 2.3 times per day on weekdays and 2.7 times per day on weekends. Three participants reported waiting to breastfeed their infant after using alcohol, one reported waiting after using cannabis, and one reported waiting after using either alcohol or cannabis. Five participants reported rarely or occasionally pumping and discarding their milk after consuming alcohol, and one reported also doing so after taking prescription medications. None reported pumping and discarding their milk after cannabis use.

**Table 2:** Cannabis use among breastfeeding individuals (*N* = 20)

**Survey questions and responses**	Mean (SD); n / N (%)
Which of the following best captures the average frequency you currently use cannabis?	
Twice a week	1 / 20 (5%)
3—4 times a week	6 / 20 (30%)
Once a day	5 / 20 (25%)
More than once a day	8 / 20 (40%)
How many days of the past week did you use cannabis?	
2 days	2 / 20 (10%)
3 days	4 / 20 (20%)
4 days	1 / 20 (5%)
5 days	1 / 20 (5%)
7 days	12 / 20 (60%)
Approximately how many days of the past 30 days did you use cannabis?	23.0 (8.6)
How many times a day on a typical weekday do you use cannabis?	2.3 (1.5)
How many times a day on a typical weekend do you use cannabis?	2.7 (1.9)
Which of the following best captures the number of times you have used cannabis in your entire life?	
11—50 times in my life	1 / 20 (5%)
51—100 times in my life	1 / 20 (5%)
101—500 times in my life	2 / 20 (10%)
501—1000 times in my life	4 / 20 (20%)
1001—2000 times in my life	1 / 20 (5%)
2001—5000 times in my life	2 / 20 (10%)
5001—10,000 times in my life	3 / 20 (15%)
More than 10,000 times in my life	4 / 20 (20%)
Prefer not to answer	2 / 20 (10%)
How many hours after waking up do you typically use cannabis?	
12—18 h after waking	3 / 20 (15%)
9—12 h after waking	5 / 20 (25%)
6—9 h after waking	2 / 20 (10%)
3—6 h after waking	5 / 20 (25%)
1—3 h after waking	4 / 20 (20%)
within 1 h of waking	1 / 20 (5%)
What is the primary method by which you use cannabis?	
Bong (water pipe)	8 / 20 (40%)
Hand pipe	1 / 20 (5%)
Joints	7 / 20 (35%)
Vaporizer (e.g., volcano, vape pen)	3 / 20 (15%)
Other	1 / 20 (5%)
What is the primary form of cannabis you use?	
Concentrates (e.g., oil, wax, shatter, butane hash oil, dabs)	8 / 20 (40%)
Marijuana (e.g., flower, bud, herb)	12 / 20 (60%)
Do you ever wait a certain amount of time after consuming a substance (e.g., cannabis, alcohol, tobacco) before breastfeeding your baby?	
Yes	5 / 20 (25%)
No	15 / 20 (75%)
If yes, after consumption of which substances do you wait to breastfeed your baby? (check all that apply)	
Alcohol	4 / 20 (20%)
Cannabis	2 / 20 (10%)
Do you ever 'pump and dump' your milk (i.e. pump your milk, then dispose of it so your baby does not consume that milk)?	
No, never	15 / 20 (75%)
Yes, occasionally	1 / 20 (5%)
Yes, rarely	4 / 20 (20%)
Do you use cannabis to treat or manage any medical or health conditions?	
No	9 / 20 (45%)
Yes	11 / 20 (55%)
Do you have a physician's recommendation to use cannabis for medicinal purposes?	
No	14 / 20 (70%)
Yes	5 / 20 (25%)
Prefer not to answer	1 / 20 (5%)
Do you have any concerns about using cannabis/marijuana while breastfeeding?	
Yes, I have a lot of concerns	1 / 20 (5%)
Yes, I have a few concerns	3 / 20 (15%)
I haven’t thought about it much	0 / 20 (0.0%)
No, I have very few concerns	12 / 20 (60%)
No, I have no concerns	4 / 20 (20%)
Have any of your healthcare providers offered advice or guidance about cannabis use while breastfeeding?	
Yes, I found it helpful	1 / 20 (5.0%)
Yes, but I did not find it helpful	5 / 20 (25%)
No, they never gave advice or guidance	13 / 20 (65%)
Prefer not to answer	1 / 20 (5.0%)

### Medical versus non-medical cannabis use

Five participants (25%) reported having a physician's recommendation to use cannabis for medical purposes. Eleven participants (55%) reported that they used cannabis to treat or manage medical or health conditions, but only two of these reported having a physician’s recommendation to do so. Only one participant who reported using cannabis for medical purposes reported exclusively medical use of cannabis while the remaining 10 participants reported a combination of medical and non-medical use. These eleven participants reported using cannabis for medical rather than non-medical purposes on average 67.3% of the time. Six participants reported only one condition that they use cannabis to treat or manage, while the other five participants listed multiple conditions. Ten out of the eleven (91%) participants reported using cannabis to treat or manage mental health conditions such as anxiety and depression, while five (45%) reported chronic pain conditions such as arthritis and back pain. Three participants (27%) reported using cannabis to treat sleep problems, and one reported using cannabis to treat psychogenic nonepileptic seizures.

**Fig. 2:**
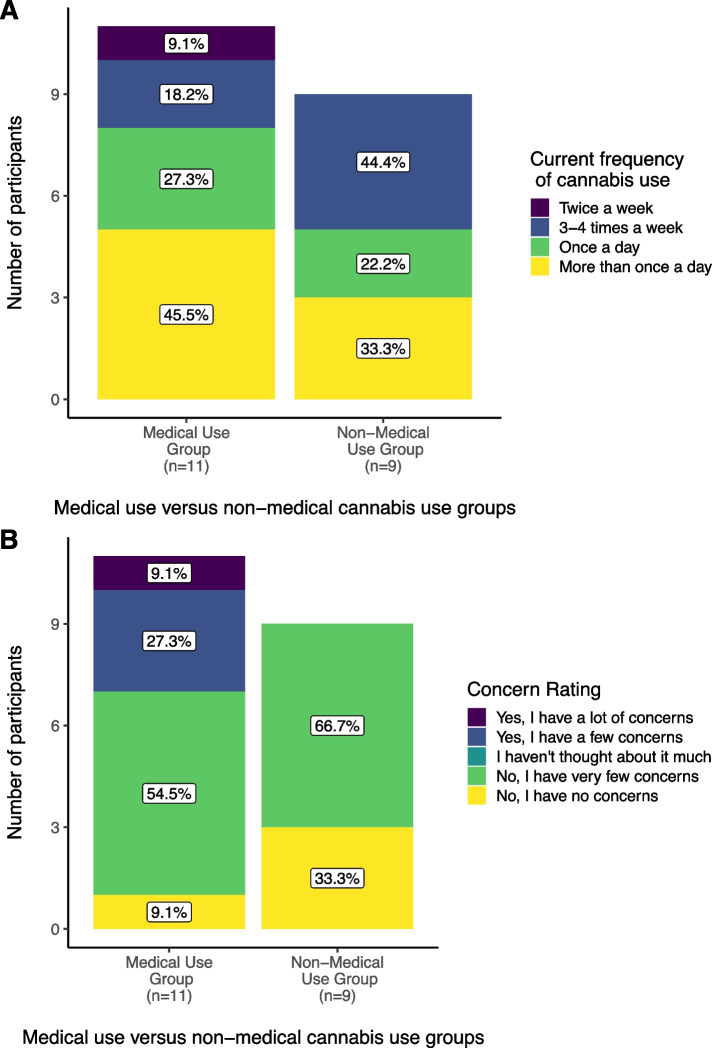
**a** Current frequency of cannabis use and **b** concerns about cannabis use, based on categorization of use as medical or non-medical (*N* = 20)

There was no difference between the medical versus non-medical groups in number of days out of the past 30 they had used cannabis (W = 51.5, *p* = 0.90), or in their current frequency of cannabis use (W = 56.5, *p* = 0.60) (Fig. 2a). Sixteen participants (80%) reported having very few or no concerns about using cannabis while breastfeeding, three reported having a few concerns, and one reported having a lot of concerns. No participants selected the response "I haven't thought about it very much." Participants using cannabis for medical purposes had significantly more concerns about using cannabis while breastfeeding than those using cannabis for non-medical purposes (medical use group mean = 3.27, non-medical use group mean = 4.33, W = 25.5, *p* = 0.04) (Fig. 2b).

### Advice from healthcare providers

Thirteen participants reported never receiving advice or guidance from a healthcare provider about cannabis use while breastfeeding (Table 2). Five participants reported they had received advice but did not find it helpful, and one reported receiving advice that they found helpful.

### Themes derived from qualitative data

Most (*n* = 14, 70%) participants elaborated about why they did or did not have concerns about using cannabis during breastfeeding. Four major themes arose from these responses: identifying research gaps and seeking out resources, monitoring child’s health and development, monitoring and titrating use, and comparing risks between cannabis and other controlled substances (Table 3). Most themes were endorsed by roughly equal numbers of medical and non-medical users, but only medical users’ responses included the theme of monitoring child’s health and development. Six responses were coded into a single theme, while the remaining eight included two or more themes, indicating that for most participants their decision to use cannabis while breastfeeding spanned multiple themes*.*

**Table 3:** Qualitative analysis of factors relating to concerns or lack of concerns (*N* = 14)

Exemplary quotes^1^	Key features of quotes by theme
**Theme 1** **: ** **Identifying research gaps and seeking out resources**	
* “I have done a lot of personal research […]”* (Participant F, has very few concerns, medical use)	Experience seeking out resources and research when making the decision to use cannabis while breastfeeding
* “From what I have read there doesn’t seem to be any harm in smoking cannabis while breastfeeding […]”* (Participant N, has very few concerns, non-medical use)	
*"I joined a Facebook group with other moms who smoke and breastfeed, and they help provide resources when anyone has questions."* (Participant L, has very few concerns, non-medical use)	
* “I know many mothers who breastfeed and smoke weed. They have healthy babies, and they are able to tend to babies’ every need in a less stressful manner, which overall, benefits the babies even more.”* (Participant D, has no concerns, non-medical use)	Use of anecdotal evidence from other people in their lives who used cannabis while breastfeeding
* “I asked a lot of people who had smoked and breastfed and they all had positive experiences and no side effects in their babies […]”* (Participant I, has very few concerns, medical use)	
* “My concern is the lack of research […] I do feel guilt concerned about it just because we don't know the long-term effects at this current time, and dosing is a large factor.”* (Participant H, has a lot of concerns, medical use)	Identified lack of research on effects of cannabis use during lactation on infant development as a reason for concern
* “There's not a lot of research out there to help me understand the way it affects my baby's development so that brings up concerns.”* (Participant G, has a few concerns, medical use)	
**Theme 2: Monitoring child’s health and development**^**2**^	
* “I have been concerned about the effect it will have on my baby’s development.”* (Participant M, has a few concerns, medical use)	Concerns related to potential effects of cannabis use on their infants’ development
* “She doesn’t seem to have any reactions when I use cannabis.”* (Participant E, has very few concerns, medical use)	Observation of their infants for any reactions to cannabis use (e.g., appetite, sleep, weight/length/growth, doctors’ feedback) as evidence that their children were not being negatively affected
*"My son is very smart, healthy, eats great, sleeps great, and is bigger than other children I know his age that are not breastfed or do not have exposure to THC.”* (Participant C, has very few concerns, medical use)	
* “I have never heard of cannabis causing issues and have never seen them in my own kids.”* (Participant A, has no concerns, non-medical use)	
* “[…] my child is doing exceptionally well according to his doctor.”* (Participant F, has very few concerns, medical use)	
**Theme 3: Monitoring and titrating use**	
* “[…] I feel safe to use it since I also don't have a high/strong use.”* (Participant E, has very few concerns, medical use)	Felt safe using cannabis due to perceived low use or purposefully reduced cannabis use to reduce risk
* “I try to limit my use due to guilt about it […]”* (Participant H, has a lot of concerns, medical use)	
* “Slight concerns only because there is not much research on the effects it has on children which is why […] I use very little amounts.”* (Participant J, has very few concerns, non-medical use)	
**Theme 4: Comparative risk between cannabis and other substances, and acknowledging costs and benefits**	
* “Cannabis is a plant.”* (Participant K, has no concerns, non-medical use)	Description of cannabis as "natural" or highlighting its status as a plant to draw distinction between cannabis and pharmaceuticals
* “I have little concern because cannabis is a natural medicine, but I’m still aware that everything including “good things” have side effects […]”* (Participant B, has very few concerns, non-medical use)	
* “[…] it truly does help with anxiety and depression. I also justify it in my head because I would rather occasionally use cannabis vs taking long term anxiety medications or benzodiazepines. I think it has less negative effects compared to some of the pharmaceuticals available. (Participant H, has a lot of concerns, medical use)*	Highlighted tradeoffs between the unknown risks of cannabis use while breastfeeding and alternative medication or no medication, concluding that the perceived benefits of using cannabis outweighed the risks
* “I […] have made the educated decision that the benefit outweighs the possible risks.”* *(Participant F, has very few concerns, medical use)*	

^1^Minor grammatical edits (e.g., spelling and punctuation) have been made to the exemplary statements above. We have included at least one quote from each participant who chose to expand on their concerns or lack of concerns, along with their level of concern and medical or non-medical use group for context

^2^While other themes were endorsed by roughly equal numbers of medical and non-medical users, only medical users’ responses were categorized into this theme, indicating that concern regarding potential developmental effects of cannabis use is particularly salient to people who use cannabis for medical reasons

## Discussion

We found that many breastfeeding individuals reported consuming cannabis for medical and non-medical purposes, and that most reported they did not receive helpful advice from healthcare providers. While most participants did not have concerns about using cannabis while breastfeeding, individuals using cannabis to treat or manage health conditions had significantly more concerns about its use. Our qualitative analysis enabled an exploration into how breastfeeding individuals approach and evaluate their decision to use cannabis. We identified four themes in participants’ responses, which combined indicate that breastfeeding individuals are engaging in self-directed assessment and are weighing the risks and benefits of cannabis use when making their decisions.

To date, few studies have explicitly explored the reasons why breastfeeding people use cannabis, and it may be these reasons, including health management, that influence the continuation, reduction, or cessation of cannabis use during breastfeeding. Our results indicate that breastfeeding individuals’ decision to use cannabis while breastfeeding is associated with multiple factors, including using cannabis to treat or manage health conditions. This finding supports previous studies that found perinatal individuals perceived cannabis to be medicinal (Barbosa-Leiker et al. 2020; Vanstone et al. 2021) and reported using cannabis for health management (Barbosa-Leiker et al. 2020; Bayrampour et al. 2019; Chang et al. 2019; Garner et al. 2022; Vanstone et al. 2021). Similar to Garner et al. (2022) the majority of our participants who defined their use as medical reported using cannabis for mental health and/or anxiety. While that study found that 89% of breastfeeding women reported using cannabis for mental or physical health symptoms (Garner et al. 2022), our participants’ rates of self-defined medical use more closely matched rates in the general population (Turna et al. 2020; Sexton et al. 2016). Understanding what health conditions breastfeeding individuals use cannabis to treat can help healthcare providers to tailor postpartum care for individuals who use cannabis.

Medical-related reasons for cannabis use may translate into differences in use patterns and therefore potential risks. Previous research has found that people who use cannabis for medical purposes report using cannabis more often than those who report only non-medical cannabis use (Lin et al. 2016; Choi et al. 2017). This is also true in breastfeeding women, with high numbers of health problems increasing the likelihood of frequent use (Garner et al. 2022). We did not find significant differences in cannabis use patterns between medical vs. non-medical users. But, given our sample size, it is possible that there are differences in cannabis use patterns between these groups that we were not powered to detect.

Most participants reported having very few to no concerns about using cannabis while breastfeeding. This finding mirrors previous findings in pregnant and non-pregnant (Barbosa-Leiker et al. 2020; Bayrampour et al. 2019; Chang et al. 2019; Cameron et al. 2022; Jarlenski et al. 2016) and postpartum individuals (Kiel et al. 2023) that cannabis is often perceived to be low risk and safer than other substances. Importantly, participants who reported using cannabis to treat or manage health conditions had significantly more concerns about cannabis use while breastfeeding than participants who reported only non-medical use. Although based on analysis from a small sample and additional research is needed, this result indicates a potential juncture where clinicians could engage with patients who rely on cannabis for their health management but are also concerned about the possible impacts. Participants in our sample as well as pregnant and postpartum participants in previous studies (Barbosa-Leiker et al. 2020) reported that the decision to use cannabis is not made uncritically—no breastfeeding individuals in our study selected the response "I haven't thought about it very much” when asked to rate their concerns.

Participants did not report legal considerations or potential investigation by agencies such as Child Protective Services to be driving their concerns about cannabis use during lactation. In previous studies, potential legal consequences emerged as a major risk perceived by women who used cannabis during the perinatal period, primarily among those who were pregnant, regardless of its legal status (Barbosa-Leiker et al. 2020; Chang et al. 2019). It may be that individuals perceive a greater legal risk of cannabis use during pregnancy than during lactation, or that those who perceived a greater risk did not participate in our study. It is also possible that such concerns characterize the six individuals in our study who participated but declined to elaborate about their concerns or lack of concerns.

Clinician counseling regarding substance use while breastfeeding is an important avenue for patient education, yet participants in our study reported receiving either unhelpful or no advice from their healthcare providers, similar to findings from other studies (Jarlenski et al. 2016; Crowley et al. 2022; Bhatia et al. 2023). A systematic review found that healthcare providers lack confidence in counseling against perinatal cannabis use due to uncertain evidence about its effects, which at times meant clinicians did not address cannabis use with patients (Panday et al. 2022). In a study that examined women’s questions about cannabis use during pregnancy and lactation posted to an online digital health platform, half of the providers discouraged use while half neither encouraged nor discouraged use (Young-Wolff et al. 2020). Patients may interpret a lack of explicit advice against cannabis use as an indication that it poses little or no risk (Bayrampour et al. 2019).

Likewise, public health messaging on cannabis use during lactation is variable, leading to confusion for breastfeeding individuals. For instance, in a content analysis of Twitter messages, Dakkak et al. (2018) found that 78% of posts about pre- and postnatal cannabis use and associated health outcomes were neutral in tone, suggesting uncertainty about the risk of cannabis use during the pre- and postnatal periods. Themes from participants’ responses indicated awareness of insufficient research, leading participants to seek out their own information. It may also be that messaging about the differential risks of commonly used substances, like alcohol and cannabis, is not clear to the public. Since alcohol concentrations in human milk closely track those in maternal blood, breastfeeding individuals are advised to wait at least two hours after a single drink to breastfeed (Reece-Stremtan et al. 2015; CDC 2022). Unlike alcohol, however, Δ9-THC is highly lipophilic (Wymore et al. 2021) and may accumulate in milk over time (Perez-Reyes and Wall 1982; Moss et al. 2021). In our sample, five participants reported waiting after consuming alcohol to breastfeed, while two reported waiting after consuming cannabis. This result provides an intriguing path for future work to assess whether people use the strategies advised to mitigate risks of alcohol consumption, without knowing that cannabinoids accumulate in human milk. As indicated by the monitoring/titrating cannabis use theme, breastfeeding individuals were cognizant of possible dose effects and attempted to mitigate possible risks rather than eliminate cannabis use entirely, consistent with similar findings in another study (Kiel et al. 2023).

### Limitations

We collected survey data from breastfeeding individuals who frequently use cannabis regarding use patterns and factors associated with their decision-making process. We recruited a sample of breastfeeding individuals with a range of reported household incomes, educational attainment, and marital status. The study’s small sample size limited our ability to identify trends in these results by other factors like race and ethnicity, to identify small effect sizes in the differences between groups, and to account for potential confounders. Recruitment through social media may have limited the scope of individuals who participated. Additionally, our qualitative analysis was based on one item that asked participants to describe why they did or did not have concerns about cannabis use while breastfeeding. Participants might have responded differently had the question been framed more neutrally but our primary goal was to understand whether participants did or did not have concerns given public health guidance regarding cannabis use while breastfeeding (Ryan et al. 2018; Committee on Obstetric Practice 2017; Reece-Stremtan et al. 2015; CDC 2022). Moreover, had longer form semi-structured interviews been collected, it would have provided the opportunity for more robust analysis. However, as this paper is a secondary analysis, we were limited by the available data.

## Conclusions

In addition to non-medical use, study participants reported using cannabis to treat or manage health conditions, especially mental health conditions. Participants used a variety of strategies and resources in their assessment of risk or lack thereof while breastfeeding, including seeking out evidence, monitoring their child’s health, titrating their cannabis use, and comparing risks between cannabis and other controlled substances. Healthcare providers, however, do not appear to be a resource that breastfeeding individuals found helpful. Most participants reported no or very few concerns about cannabis use while breastfeeding, although individuals who use cannabis for medical purposes may have more concerns about its use. For medical providers to advise on best practices, they must be aware of the reasons people choose to use cannabis while breastfeeding, as well as the reasons why concerns about use are low.

## Data Availability

The datasets generated and/or analyzed during the current study are not publicly available due to participants’ privacy.

## Abbreviations

US: United States
Δ9-THC: Delta-9-tetrahydrocannabinol
CBD: Cannabidiol
GED: General Educational Development
DFAQ-CU: Daily Sessions, Frequency, Age of Onset and Quantity of Cannabis Use Inventory
